# Biocomputational analysis of evolutionary relationship between toll-like receptor and nucleotide-binding oligomerization domain-like receptors genes

**DOI:** 10.14202/vetworld.2016.1218-1228

**Published:** 2016-11-10

**Authors:** Rabia Bhardwaj, Chandra Shekhar Mukhopadhyay, Dipak Deka, Ramneek Verma, P. P. Dubey, J. S. Arora

**Affiliations:** 1 School of Animal Biotechnology, Guru Angad Dev Veterinary and Animal Sciences University, Ludhiana, Punjab, India; 2 Department of Animal Genetics and Breeding, Guru Angad Dev Veterinary and Animal Sciences University, Ludhiana, Punjab, India

**Keywords:** bioinformatics, domain, evolution, nucleotide-binding oligomerization domain-like receptors, selection pressure, toll-like receptor

## Abstract

**Aim:**

The active domains (TIR and NACHT) of the pattern recognition receptors (PRRs: Toll-like receptors [TLRs] and nucleotide-binding oligomerization domain [NOD]-like receptors [NLR], respectively) are the major hotspots of evolution as natural selection has crafted their final structure by substitution of residues over time. This paper addresses the evolutionary perspectives of the TLR and NLR genes with respect to the active domains in terms of their chronological fruition, functional diversification, and species-specific stipulation.

**Materials and Methods:**

A total of 48 full-length cds (and corresponding peptide) of the domains were selected as representatives of each type of PRRs, belonging to divergent animal species, for the biocomputational analyses. The secondary and tertiary structure of the taurine TIR and NACHT domains was predicted to compare the relatedness among the domains under study.

**Results:**

Multiple sequence alignment and phylogenetic tree results indicated that these host-specific PRRs formed entirely different clusters, with active domains of NLRs (NACHT) evolved earlier as compared to the active domains of TLRs (TIR). Each type of TLR or NLR shows comparatively less variation among the animal species due to the specificity of action against the type of microbes.

**Conclusion:**

It can be concluded from the study that there has been no positive selection acting on the domains associated with disease resistance which is a fitness trait indicating the extent of purifying pressure on the domains. Gene duplication could be a possible reason of genesis of similar kinds of TLRs (virus or bacteria specific).

## Introduction

Early sensing followed by immediate dismissal of pathogenic microbes by the host immune system is the primary mechanism of host defense mechanism. The innate immune response initiation requires recognition of conserved, molecular structures broadly shared by microbes, and pathogens that are known as pathogen-associated molecular patterns (PAMPs) and is mediated by germline-encoded pattern recognition receptors (PRRs) [1]. On detection of PAMP within the host’s body, PRRs trigger intracellular signaling cascades that execute the first line of host defense through the expression of a variety of pro-inflammatory molecules leading to maturation of dendritic cells, responsible for the subsequent activation and shaping of adaptive immunity, thereby bridging the two arms of immunity. Two families of PRRs, *viz*., Toll-like receptors (TLRs) and nucleotide-binding oligomerization domain (NOD)-like receptors (NLRs) are evolutionary conserved from early invertebrates to recent mammals. These receptors have direct involvement in fitness of the individuals by conferring resistance against various microbes [2].

TLRs were the first type of PRRs identified that perceive an extensive variety of PAMPs. Till date, more than 10 functional TLRs have been identified. TLRs recruit a specific set of adaptor molecules that harbor TIR domain, such as MyD88 and TIR-domain-containing adapter-inducing interferon-β. NLR family includes more than 20 NLRs that communicate intracellularly and respond to various PAMPs to trigger inflammatory responses. One such study provided with evidence of TLR being able to orchestrate immediate global and local responses through both quantitative and qualitative cytokine production, thereby leading to generation of highly specific innate immune response [3]. A few NLRs, for example, NALP1 (NLRP1) and NALP3 (NLRP3), those having the NACHT domain structure the inflammasomes alongside ASC and caspase-1 complexes, intercede preparing of mature IL-1b from pro IL-1, resulting in mobilization of the effectors of inflammation, which is another pathway for innate immune response elicitation [4]. TLRs, working together with NLRs, act in a synergistic, complementary, or compensable manner to orchestrate proficient elimination of pathogenic bacteria [5]. TIR and NACHT are the important domains of the TLR and NLR families, respectively, which are the most studied, potent mediators of inflammation. Evolution and functional specification of protein domains reveal the evolutionary relics of the conserved patterns, and their studyis relatively more important than the complete gene study because domains are the conserved part of protein sequences which has evolved for functional diversification than rest of the protein chain. Thus, in the light of evolutionary study, domains particularly provide an insight of how molecular evolution uses domains as building blocks of protein architecture over the years. Proell and team performed the similar work, where the sequence and structure modeling details of various NLRs, where evolutionary tree depicted the conservation of characteristic functional regions within the NLR family [6]. Analysis of NACHT domains was done rather than full-length protein, to address the possible evolutionary history of NLR proteins. Specific functional roles of the leucine-rich repeat (LRR) domains of TLR and NLR genes has already been demonstrated by performing quantitative and comparative analysis of surface features of LRR domains in humans. Comparison of LRR surface features led its way to the possible hypothesis of similar functional roles despite the differences in structural and genomic organization [7]. Zhang *et al* estimated that the evolution of TLR and NLR families took place in a species specific as well as domain shuffling manner, which predicted the evolution of same domain architectures, independently [2].

A number of reports are available on the evolution of domain architecture as well as functional roles of many PRR domains, but till date, the evolutionary relationships between TLR and an NLR domain is largely unknown. Thus, our present research work targets the determination of evolution of the PRR domains (TIR and NACHT domains of TLR and NLR genes, respectively) in divergent animal species with regard to the impact of natural selection, their chronological divergence and structural attributes implying the functional specification.

## Materials and Methods

### Ethical approval

There is no need to obtain permission from institutional animal ethics committee to pursue this type of study.

### Sequence database searches

The available TLRs and NLRs full-length cds and amino acid sequences of divergent species were downloaded in FASTA format from nucleotide database of NCBI for studying the evolutionary relationship between different species. The TIR and NACHT domains within the TLR and NLR peptides, respectively, were identified using SMART database (http://smart.embl-heidelberg.de/), and the domain-specific coding sequence (open reading frames [ORF]) and the translated amino acid sequences were saved in FASTA format in separate files for further analysis.

### Construction of phylogenetic tree and time tree

The domain specific nucleotide sequences of divergent TLRs and NLRs from various animal species were subjected to multiple sequence alignment using online tool multiple alignment using fast Fourier Transform (http://www.ebi.ac.uk/Tools/msa/mafft/). The ORF of coding sequences weresubjected to evolutionary analyses. The best evolutionary model was determined based on the least Bayesian information criterion (BIC) scores using MEGA6 [8]. The Akaike Information Criterion, corrected (AICc) value, maximum likelihood value (lnL), and the number of parameters (including branch lengths) were compared for each of the models.

The coding sequences were subjected to analysis using maximum likelihood method for the phylogenetic tree construction, and the reliability of the branching of the tree was checked by 1000 bootstrap resampling [9]. Construction of phylogenetic tree, Fisher’s exact test and codon-based test for determining the selection pressure on the domains was done using MEGA6 software [8].

The molecular clock test was performed on the divergent PRR-domains against the null hypothesis of equal evolutionary rate by comparing the maximum likelihood values for the given topology with and without the molecular clock constraints under general time reversible (GTR) model along with gamma distributed rates among sites with 5 discrete gamma categories [10]. Divergence time calibration constraints of minimum and maximum divergence time as 1 and 10 units, respectively, to optimize and convert into absolute divergence time. The log-likelihood value of the used topology (to calculate the divergence time of each of the RelTime tree) is −13,170.36. The branch length of the tree has been adjusted to the relative number of substitution per site.

### Estimation of evolutionary divergence and homogeneity of substitution patterns

The evolutionary divergence between all the selected 48 coding sequences for TLR and NLR domains were estimated to obtain the base substitution per site using the maximum composite likelihood model, with gamma distribution of 5 categories [11]. The heat map was constructed using data generated by the analysis of relative pairwise distances between the PRR domains by WGCNA package of R (version 3.2.3). Codon-based test of purifying selection for analysis between sequences has been performed using the probability of rejecting the null hypothesis of strict neutrality (H_0_: dN = dS) in favor of the alternative hypothesis (H_1_: dN<dS). Here, “dS” and “dN” are the numbers of synonymous and nonsynonymous substitutions per site, respectively. The variance of the difference between the substitutions was computed using the analytical method using the Nei-Gojobori method. The analysis for codon-based test of purifying selection for analysis between sequences indicated that the null hypothesis of strict neutrality hasbeen clearly rejected in the species with p<0.05.

### Determining the sites of positive or negative selection

The extent of positive selection by specific codons wasdetermined by Datamonkey (www.datamonkey.org/) online server, using different statistical methods, single likelihood ancestor counting (SLAC), fixed effects likelihood (FEL), Internal branch FEL, and random effects likelihood (REL). A hierarchical testing was done with nested likelihood ratio tests along with AIC selection (http://www.datamonkey.org/help/models.php). Variation in rate of evolution along both branches, and sites was adjusted by Branch-site REL tests [12] for episodic diversifying selection. This analysis enables to determine the lineages on which a subset of sites has evolved under positive selection, without requiring prior knowledge about which lineages are of interest.

### Comparison of predicted structures of domains

The predicted amino acid sequences (using ExPASy translation tool: http://web.expasy.org/translate/) of the TLR and NLR domains were subjected to secondary structure prediction using PSIPRED v3.0(http://bioinf.cs.ucl.ac.uk/psipred/) and *ab initio* tertiary structure prediction using online tools RaptorX (http://raptorx.uchicago.edu/). The three-dimensional (3D) protein structure obtained from RaptorX was subjected to structure validation using WHAT IF online tool (http://swift.cmbi.ru.nl/whatif/), and Ramachandran’s plot analysis was performed for validation of the predicted protein structure using molprobity online tool (http://molprobity.biochem.duke.edu/).

## Results

### Multiple sequence alignment and phylogeny construction

**Figure-1:**
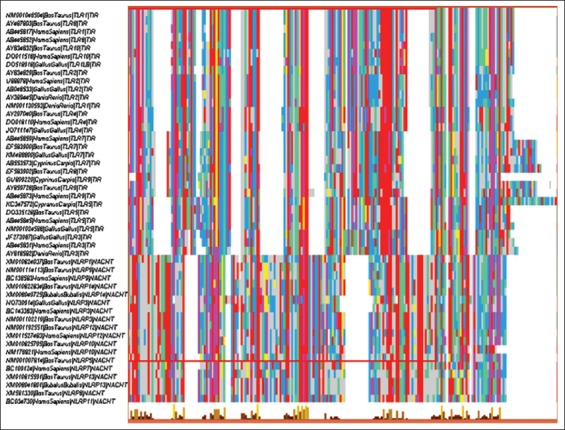
Multiple sequence alignment overview window (generated by multiple alignment using fast Fourier transform online tool) displaying the alignment of cds encoding active domains of divergent toll-like receptors and Nod like receptors from various animal species.

**Figure-2:**
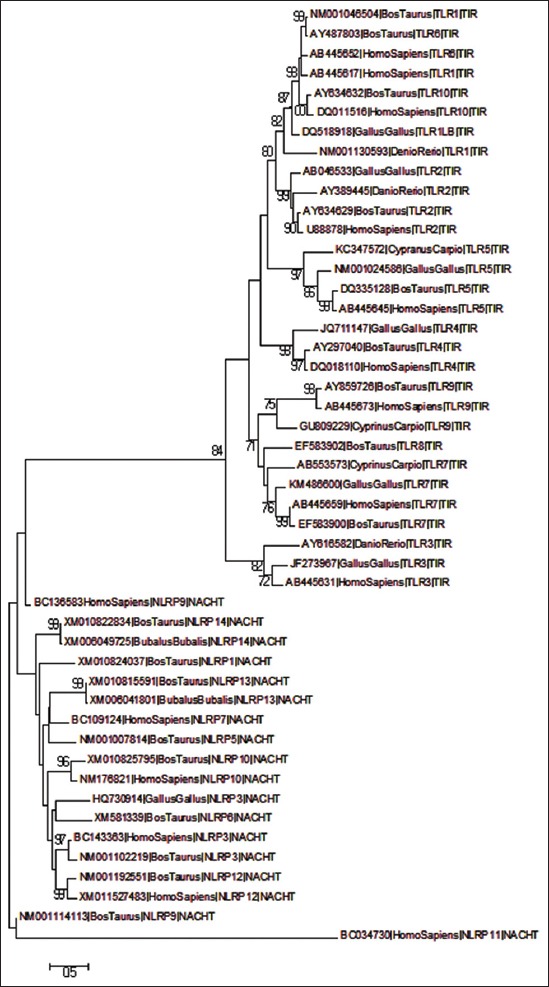
Phylogenetic tree constructed from cds encoding the active domains of 48 divergent toll-like receptors and Nod like receptors, using maximum likelihood method (1000 bootstrap resampling).

The multiple sequence analysis was performed on the coding sequences of TIR domains of TLR and NACHT domains of NLRs of divergent species. The overall alignment window (shown below) was saved to depict the conserved regions of all TIR and NACHT domains (Figure-1). The best model, i.e. GTR+gamma (G) was selected for further evolutionary analysis selected cds depicted that GTR+G was the best model among the rest with minimum BIC score of 27,381.45. The phylogenetic tree was constructed subjecting 48 TLR and NLR coding sequences, each representing a set of divergent class/order of animals for comprehensible interpretation (Figure-2).

The phylogenetic tree revealed the separate branching of viral-recognizing TLRs (3, 7, 8 and 9) from that of non-virus-specific TLRs (1, 6 and 10). The later set of TLRs was forming a separate branch at the top of the phylogenetic tree, whereas former TLRs were clustering near the TLR3 sub-branch. In another study predicted by Areal *et al* worked on mammalian TLR domains (LRR, TIR, etc.) and studied to identify signatures of positive selection, and the results indicated that viral(TLR3, 7, 8, 9) and non-viral (TLR1-6, 10) TLRs display different patterns of molecular evolution [13]. NLRP11 forms a separate node from rest of the NLRs and other NLRs which form several different sub-branches within a major branch. The phylogenetic tree depicts even the clustering of various NLRs according to their functional divergence, being reproduction (NLRP 2, 4 and 7) and non-reproduction related (3, 6, 10 and 12). Tian *et al* evaluated the evolution and functional divergence of NLRP genes depicting that NLRP11 being a primate-specific NLR undergoes lineage-specific duplication in primates [14].

**Figure-3:**
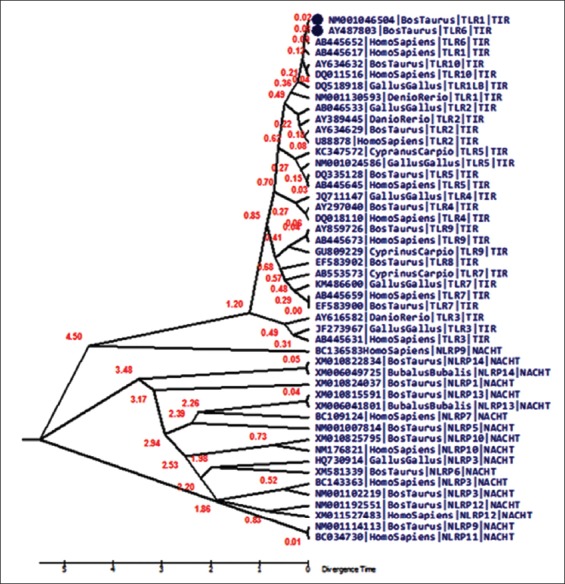
Molecular clock tree depicting the divergence of the pathogen recognition receptor-domains over time. The tree has been constructed using molecular clock test to compare the maximum likelihood values for the tree topologies obtained assuming presence vis-a-vis absence of the molecular clock constraints under general time reversible model (+G).

The molecular clock test phylogenetic tree divergence time depicted in the clock test was in agreement to the traditional evolution, wherein each of the TLRs and NLRs branched out in a specific manner of fishes being the first to branchout, followed by avian and lastly mammals (Figure-3).

### Estimation of evolutionary divergence and homogeneity of substitution patterns between sequences

**Figure-4:**
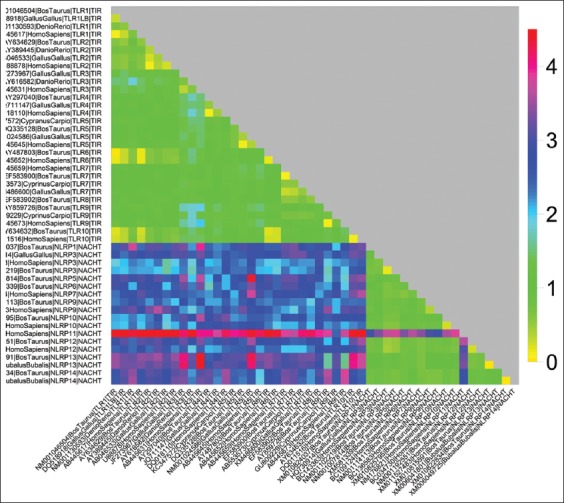
Evolutionary divergence heat map: Showing the relative distances among the coding sequences of 48 toll-like receptors and Nod like receptors domain belonging to different animal species.

**Figure-5:**
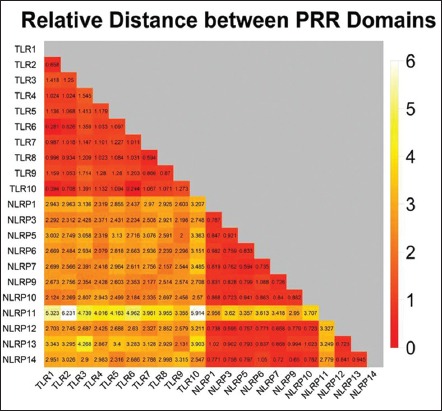
Evolutionary divergence heat map: Showing the relative distances of sequence pairs between groups of various PRR domains (constructed by taking mean from each group of divergent animal species).

Evolutionary divergence was calculated based on the prediction of heat maps. The heat map is a colored graphical representation of the evolutionary divergence of the TLRs and NLRs coding sequences in different species incorporated in this study. The heat map showed that evolutionary divergence (Figure-4) was maximum in the NACHT domains of NLRP11 (magenta) while least evolutionary divergence, i.e., maximum conservation was seen in TIR and NACHT domains of TLRs and NLRs (red) with respect to each other. Intermediary evolutionary divergence (green) was noted in the case of NLR domains with respect to various TLR domains. The mean distance between each PRR-group was calculated which have been depicted as heat map (Figure-5), which shows that the least distance (red) was seen among TLRs and NLRs. When considered in respect to the evolutionary divergence between TLR and NLRs, the distance was intermediary (yellow). The values of divergence within the same group of various PRR discussed shows that TLR5 and NLRP3 show maximum values for base substitution per site (Table-1).

**Table 1:** Estimates of average evolutionary divergence over sequence pairs within groups.

Groups	Base subst/site*	SE
TLR1	0.43	0.04
TLR2	0.29	0.03
TLR3	0.53	0.05
TLR4	0.37	0.04
TLR5	0.49	0.04
TLR6	0.14	0.02
TLR7	0.39	0.04
TLR8	n/c	n/c
TLR9	0.55	0.06
TLR10	0.13	0.02
NLRP1	n/c	n/c
NLRP3	0.49	0.05
NLRP5	n/c	n/c
NLRP6	n/c	n/c
NLRP7	n/c	n/c
NLRP9	0.37	0.04
NLRP10	0.27	0.03
NLRP11	n/c	n/c
NLRP12	0.25	0.03
NLRP13	0.02	0.01
NLRP14	0.01	0.01

* Base substitution per site. SE=Standard error, TLR=Toll-like receptor, NLR=Nod like receptor

### Analyzing the positive and negative sites

**Figure-6:**
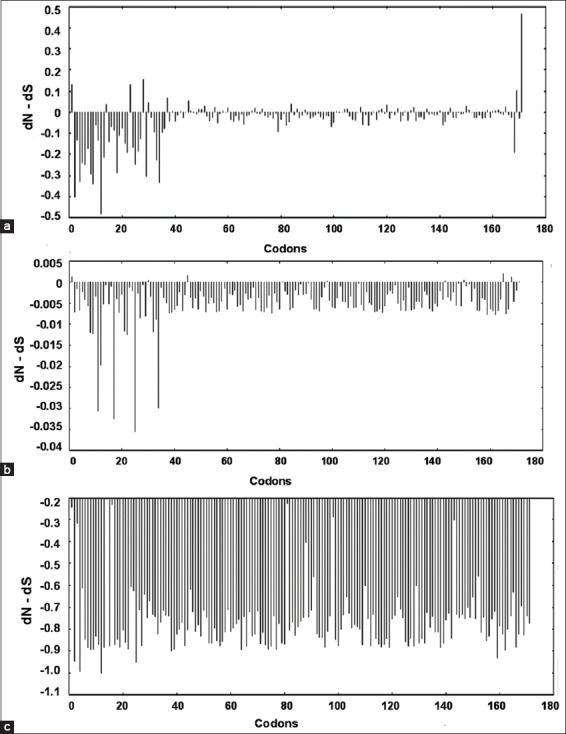
Graphical representation of the dN-dS test statistic versus the codon positions obtained from (a) Single likelihood ancestor counting, (b) fixed effects likelihood, and (c) random effects likelihood analyses, predicting mostly the negatively selected sites.

Datamonkey results for 48 representative divergent sequences were retrieved using different models, namely SLAC, FEL, and REL. The SLAC revealed 6 positively and 49 negatively selected sites. The results for FEL and REL model predicted only negatively selected sites, thus suggesting that all sites are under purifying selection. The graph plot of SLAC FEL and REL is presented in Figure-6.

**Figure-7:**
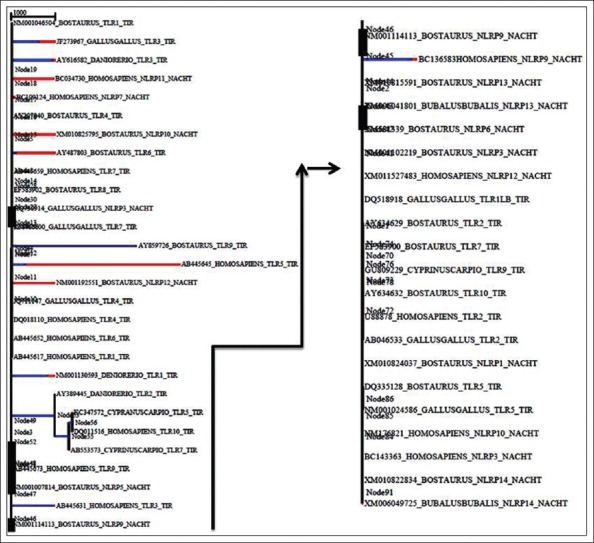
Phylogenetic tree of 48 divergent animal species based on BS-random effects likelihood result depicting the episodic diversifying selection. The colors of the branches signify strength of selection: Blue corresponds to purifying selection (ω=0), black or grey to neutral or nearly neutral (ω=1), and red color corresponding to diversifying (or positive) selection (ω>5) while the width of the branch corresponds to the proportion of sites undergoing episodic diversifying selection (for better representation, the tree has been halved and arranged horizontally).

Branch site REL scaled on the expected number of substitutions/nucleotide is given in the Figure-7. The colors of thebranches of the tree signify strength of selection: Blue corresponds to purifying selection (ω=0), black or gray to neutral or nearly neutral (ω=1), and red color corresponding to diversifying (or positive) selection (ω>5) while the width of the branch corresponds to the proportion of sites undergoing episodic diversifying selectionby the sequential likelihood ratio test at corrected p≤0.05. Branch site REL analysis results revealed 6 nodes under episodic diversifying selection at 5% (p≤0.05).

### Comparative analysis of protein structure prediction

**Figure-8:**
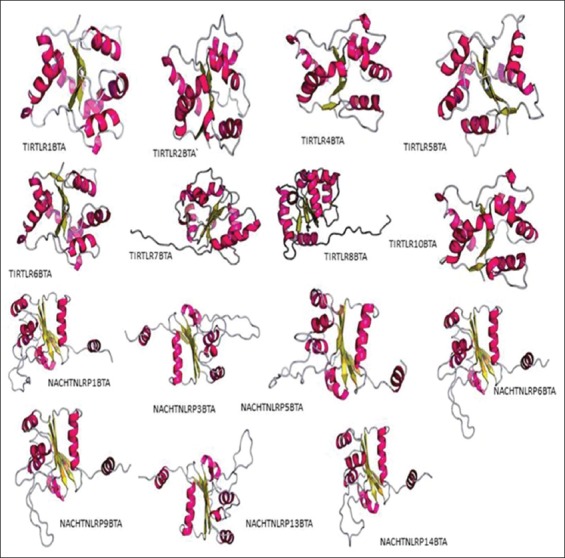
Three-dimensional structures of TIR and NACHT domains of taurine toll-like receptors and nucleotide-binding oligomerization domain like receptors (*ab initio* method) using the software Raptor X, showing the exact positioning of α-helices and beta pleated sheets.

Protein structure prediction analysis was performed for 11 PRR-domain specific sequences of *Bos taurus*. The structure of NLRP10 of the species could not be used for structure prediction due the limitations imposed by the in-between stop codon (at 24^th^ and 143^rd^ codons) in the original sequence (cds: XP010824097, GenPept: 104974255). Protein secondary structure prediction revealed that NACHT domains were longer as compared to the TIR domains. Secondary structure prediction of the sequences revealed the number of sheets and helixes among different types of TLR and NLR domains in taurine species. An almost same number of sheets were present in all the TIR domains and a clear excess of helix in NACHT domains as compared to that of TIR domains were also observed. The absence of coiled coil structure was seen in almost all the sequences. The 3D protein structure analysis showed beta pleated sheets, α-helix and loop structures. Ramachandran plot validation revealed more than 90% of the structure in favoredregions and 95-100% of the structure is lying in allowed regions (Table-2). 3D predicted structures from RaptorX are shown in Figure-8. Visual description of the Raptor X tertiary structures predict the presence of long but few α-helices in TIR domains as compared to the NACHT domains which constitute shorter but more number of α-helices. An almost equal number of beta pleated sheets was seen in both TIR and NACHT domains, but the structure prediction clearly predicts the positioning of these sheets in the center of the structure, indicating their hydrophobic nature. The absence of coils in both the TIR and NACHT domains was also confirmed by both the secondary as well as tertiary structure prediction results. Howe and coworkers analyzed the NLRs gene family in zebrafish and studied its evolutionary history. The protein structure prediction revealed overall structure similarity, but individual domains show different evolutionary patterns [15]. Structure prediction of bubaline Drosha enzyme was done using similar protein structure prediction tools and revealed that in light of evolution prokaryotic RNases have evolved according to the functional requirements [16].

**Table 2:** Secondary structure prediction results.

TLR and NLR domains and species	^#^H-S-C^$^	Raptor Xp value	^#^Amino acids^@^	Number of residues in the regions		

				Favored* (%)	Allowed** (%)	Outlier
TIRTLR1Bta	5-5-0	3.85e-08	142	125 (88.0)	141 (99.3)	1
TIRTLR2Bta	5-5-0	1.38e-07	143	134 (93.7)	141 (98.6)	2
TIRTLR4Bta	5-5-0	1.48e-06	144	132 (91.7)	144 (100.0)	0
TIRTLR5Bta	5-5-0	1.99e-07	144	138 (95.8)	143 (99.3)	1
TIRTLR6Bta	5-5-0	5.46e-08	142	139 (97.9)	142 (100.0)	0
TIRTLR7Bta	5-4-0	1.51e-06	157	147 (93.6)	154 (98.1)	3
TIRTLR8Bta	5-5-0	1.04e-06	159	150 (94.3)	157(98.7)	2
TIRTLR10Bta	5-5-0	3.74e-08	144	137 (95.1)	141 (97.9)	3
NACHTNLRP1Bta	8-4-0	3.42e-07	168	153 (91.1)	162 (96.4)	6
NACHTNLRP3Bta	7-5-0	6.09e-07	168	156 (92.9)	163 (97.0)	5
NACHTNLRP5Bta	7-4-0	5.74e-07	163	147 (90.2)	156 (95.7)	7
NACHTNLRP6Bta	6-5-0	3.79e-07	168	154 (91.7)	163 (97.0)	5
NACHTNLRP9Bta	7-4-0	3.79e-07	168	156 (92.9)	164 (97.6)	4
NACHTNLRP10Bta	6-4-0	NA*	NA*	NA*		
NACHTNLRP12Bta	6-5-0	4.02e-07	169	159 (94.1)	166 (98.2)	3
NACHTNLRP13Bta	7-5-0	2.81e-07	168	158 (94.0)	165 (98.2)	3
NACHTNLRP14Bta	7-4-0	3.70e-07	168	153 (91.1)	164 (97.6)	4

$ H=Helix, S=Beta pleated sheet, C=Coils.

* The prediction could not be done due to presence of stop codons (at 24^th^ and 143^rd^ codons) in the original predicted coding sequence (cds: XP010824097, GenPept: 104974255),

@ Total number of amino acids of each of the corresponding TIR and NACHT domains,

* Against expected number of residues in favored region for all the sequences as revealed by Ramachandran’s plot analysis,

** Against expected number of residues in allowed region for all the sequences as revealed by Ramachandran’s plot analysis

## Discussion

Phylogenetic tree indicated that TLR and NLR genes have undergone natural selection through the time in accordance with the requirement of environment. The evolutionary tree clearly indicated distinct clustering of TLRs and NLRs, with TLRs forming an entirely different branch from those of NLRs. The phylogenetic tree on a broader perspective depicted that the taurine TLR and NLR sequences are closely related to that of humans, both being the mammalian species, while the avian and piscine form a separate node. Huang and co-workers cloned the TLR7 gene of Chinese Salamander, where the phylogenetic analysis predicted the clustering of avian and reptiles apart from those of mammals and piscine supporting the traditional understanding of animal evolution [17]. Alcaide and team also worked on the molecular evolution of TLR domains in divergent non-model avian species and demonstrated the first ever model of avian evolutionary pattern as well as TLR polymorphism, which indicated a huge excess of silent nucleotide substitution, in agreement to our current work [18].

Furthermore, the TLRs are shown to make separate branches depending on the use of different pathways used by different TLRs. TLR3 diverging into an entirely different branch from the rest of the TLRs as it utilizes the MyD88 independent pathway, unlike the rest of the TLRs, which makes use of MyD88 dependent pathway. A similar finding was supported by Roach *et al*. which demonstrated that TLR can be grouped depending on the usage of the MyD88adaptor. They presented a unique phylogenetic analysis of TIR domain regions of TLR receptors indicating a separate evolutionary origin for the MyD88-dependent and MyD88-independent signaling pathway [19].

The clock test for TLR sequences revealed a clear-cut divergence time difference between TLRs and NLRs, which further evolved over time. As can be seen in the divergence tree TLR3 is forming a separate clade from the rest of the TLRs. TLRs and NLRs evolved separately with *Homo sapiens* NLRP9 branching out from the TLR clad at a later interval. Human and taurine TLRs diverged rather recently as compared to what was seen in NLRs. Overall, molecular clock test revealed a recent divergence of TLRs as compared to NLRs, as NLRs were seen to separate out quite early in evolution.

Gene duplication is an important event of evolutionary process, where duplicates are produced to create new functions during functional divergence and process of speciation [20]. Molecular clock test phylogenetic tree describes the common ancestral relationship between TLRs and NLRs but with functional diversification, they both separated out during a major divergence event at an early stage of evolution. Huang *et al* predicted the molecular evolution pattern in TLR1 gene of birds and mammals and demonstrated that TLR1 gene family has been mostly under purifying selection, with few sites of directional selection as well [21]. Comparative phylogenetic analysis of chicken TLR receptor gene family was performed by Temperley *et al.*, proving insights of gene evolution. Phylogenetic analysis shoes the absence of TLR8 and TLR9 in chicken, due to gene loss event [22]. NLR genes have many a times undergone extensive expansions and fusion phenomenon as well as duplication event independently in many species throughout evolution [15].

Evolutionary divergence measures the divergence of sequences between two species with respect tocertain parameter. The cluster formation in heat maps also provided with the same inference of NLRP11 being the divergent of all. NLRP11 being a primate-specific NLR was not a part of clusters made by NLR family rather was diverging separately from all in the form of a separate branch. The divergence within same group of various PRR shows that TLR5 and NLRP3 shows maximum values for base substitution per site and is being supported by another study of TLR molecular evolution study in avain TLRs, TLR5 was shown to be exhibiting highest levels of genetic polymorphism and nonconservative amino acid substitutions, in agreement with our search results [18].

The codon-based test predicted the clear excess of synonymous mutation (dS) as compared to nonsynonymous mutation (dN) predicting the purifying selection. A similar study of the sequence analysis of the TLR4 gene in *Bubalus bubalis* indicated that the sequences (ruminants) experienced a purifying selection during the course of evolution as the null hypothesis of strict neutrality in favor of the alternative hypothesis of positive selection was rejected [23]. In another recent study of *in silico* characterization and functional divergence of two cathelicidin variants in sheep also suggested the separate clustering of mammalian cathelicidins from avian fowlicidins. Avians showed a purifying selection pattern quite different from what was seen in mammalian [24].

The graph plot of SLAC REL and FEL is a diagrammatic representation of dN-dS values versus the codons. It can be concluded that there are very few positively selected sites in SLAC model and none found in both FEL and REL analyses, depicting only negative/purifying selection. Hence, all the codons have experienced purifying selection during evolution. Purifying selection in Avian TLRs was seen in the prediction of evolution patterns of TLR multi-gene family. A clear excess of synonymous over nonsynonymous at every TLR locus was seen, using SLAC and REL models with positive selection was seen only in few pathogen recognition domains [18].

A branch-site evolutionary model provides a framework, based on statistical estimation to provide adequate evidence for episodic selection. Branch-site tests measure selective pressure by estimating the value of omega (ω), i.e. the ratio of non-synonymous (β) to synonymous (α) substitution rates, and the episodic positive selection is inferred if the proportion of sites in the sequence gives statistically significant support for ω>1 along the lineages. The phylogenetic tree based on branch-site REL result depicting the episodic diversifying selection. It was seen on most lineages, when a site evolves under purifying selection, site methods which assume ω is constant over time may be unable to identify any episodic positive selection. It has been noted that positive selection is more readily identified in smaller alignments because inclusion of additional sequences may cause no longer detection of sites [12]. In another study, using the same data monkey models, bubaline Dicer I gene was studied to be undergoing purifying selection. Comparative analysis revealed the conservation of domain structure across all the higher eukaryotic species [25].

## Conclusion

This study predicts evolutionary pattern of TIR and NACHT domains of the TLR and NLR genes, respectively. Molecular phylogeny clearly depicts similar patterns of evolution in divergent species (mammalian, avian and piscine); however, different evolutionary time frame (NLRs evolved earlier as compared to TLRs) is evident for these two domains under study. Interestingly, only negative selection (indicating purifying pressure) has been impacting the domains of TLR and NLR genes. The domains are associated with disease resistance which is a fitness trait. Each group of TLR or NLR shows comparatively less variation within themselves again due to the specificity of action against the type of microbes. It can be proposed that gene duplication could be a possible reason of genesis of similar kinds of TLRs (virus-specific, bacteria specific).

## Authors’ Contributions

CSM: Conceived and designed the experiments. CSM, RB, DD and RV: Analyzed the data. RB: Wrote the first draft of the manuscript. JSA and PPD: Contributed to the writing of the manuscript. RB, CSM, DD, RV, PPD and JSA: Agree with manuscript results and conclusions. RB, CSM, DD, RV, PPD and JSA: Jointly developed the structure and arguments for the paper. RB, CSM, DD, RV, PPD, JSA: Made critical revisions and approved final version. All authors reviewed and approved the final manuscript.
